# Supplementary material for: Molecular and Clinical Aspects of Osteogenesis Imperfecta Type VI: A Case Series with Novel SERPINF1 Gene Variants
Source: Int J Mol Sci. 2025 Jun 27;26(13):6200. doi: 10.3390/ijms26136200 (PMC12250282; doi:10.3390/ijms26136200)
Supplement: Supplementary file 1 [file ijms-26-06200-s001.zip › ijms-3644311-supplementary.pdf]

Supplementary Material

Title: Molecular and Clinical Aspects of Osteogenesis Imperfecta Type VI: A Case Series with Novel *SERPINF1* Gene Variants

Authors:

Elena S. Merkuryeva[1]*, Tatyana S. Nagornova[1], Vladimir M. Kenis[2], Anna S. Deviataikina[1], Daria B. Akimova[1], Dmitry S. Buklaev[2], Ilya S. Dantsev[3], Aisluu O. Dulush[4], Ekaterina Y. Zakharova[1], Tatiana V. Markova[1]

Affiliations:

[1] Research Centre for Medical Genetics, 115522 Moscow, Russia

[2] The Turner Scientific Research Institute for Children's Orthopedics, 196603 Saint Petersburg, Russia

[3] Veltischev Research and Clinical Institute for Pediatrics, Pirogov Russian National Research Medical University, 125412 Moscow, Russia

[4] Perinatal Center of the Republic of Tuva, 667000 Kyzyl, Russia

*Corresponding author: elena.merkureva@gmail.com

Supplementary Material

Gene List of the Panel 'Hereditary Diseases with Skeletal Pathology' (166 genes)

ADAMTSL2, AGPS, ALPL, ANKH, ARSB, ARSE, ATP6V1B1, ATP6V0A4, BMP1, CA2, CLCN5, COL10A1, COL11A1, COL11A2, COL1A1, COL1A2, COL2A1, COL9A1, COL9A2, COL9A3, COMP, CREB3L1, CRTAP, CTSA, CYP27B1, CYP2R1, DDR2, DMP1, DYM, DYNC2H1, EBP, EHHADH, ENPP1, EVC, EVC2, FAH, FBN1, FGF23, FGFR3, FKBP10, FLNA, FLNB, FUCA1, GALNS, GLB1, GNPAT, GNPTAB, GNPTG, GNS, GPX4, GUSB, HGSNAT, HNF4A, HSPG2, IDS, IDUA, IFITM5, IFT122, IFT140, IFT172, IFT43, IFT80, IL1RN, INPPL1, LBR, LEPRE1, LIFR, LONP1, LRP5, MAN2B1, MANBA, MATN3, MCOLN1, MMP13, MMP9, NAGLU, NEK1, NEU1, NME1, NKX3-2, NSDHL, PAPSS2, PEX1, PEX5, PEX6, PEX7, PHEX, PHOSPHO1, PLOD2, PLS3, PPIB, PTH1R, RAB33B, RUNX2, SBDS, SERPINF1, SERPINH1, SGSH, SLC17A5, SLC26A2, SLC2A2, SLC34A1, SLC34A3, SLC35D1, SLC4A1, SLC4A4, SMARCAL1, SOX9, SP7, SPARC, SPP1, SUMF1, TCTN3, TMEM38B, TRIP11, TRPV4, TTC21B, VDR, WDR19, WDR34, WDR35, WDR60, WNT1, RMRP, FBN2, MYH3, ROR2, BTK, GDF6, GNAS, MSX2, TRIM37, TCIRG1, GLI3, FGFR1, FGFR2, HOXD13, ROBO3,

DLL3, TRAPPC2, ACVR1, ERCC6, HEXB, HEXA, HYAL1, GM2A, AP2S1, CASR, GNA11, SLC9A3R1, CLCN7, GJA1, HPGD, MTAP, OSTM1, PLEKHM1, PTDSS1, SLCO2A1, SNX10, SOST, TBXAS1, TGFB1, TNFRSF11A, TNFRSF11B, TNFSF11, TYROBP.

Table S1. Public genetic databases used in the study

Name (Abbreviation)	Full Name	URL	Description
OMIM	Online Mendelian Inheritance in Man	https://www.omim.org/	Catalog of human genes and genetic disorders, including phenotypes and gene-phenotype relationships.
LOVD	Leiden Open Variation Database	https://www.lovd.nl/	Open-source gene variant database for sharing and accessing genetic variants.
gnomAD	Genome Aggregation Database	https://gnomad.broadinstitute.org/	Aggregated population-level sequencing data for estimating

			variant frequencies.
1000 Genomes Project	—	https://www.internationalgenome.org/	Global reference dataset of common human genetic variation.
ESP6500	NHLBI Exome Sequencing Project	https://evs.gs.washington.edu/EVS/	Variant frequency data from 6,500 exomes from multiple populations.
HGMD Professional	Human Gene Mutation Database	https://www.hgmd.cf.ac.uk/	Commercial database of known gene mutations associated with human inherited diseases.
